# In Black South Africans from Rural and Urban Communities, the 4G/5G PAI-1 Polymorphism Influences PAI-1 Activity, but Not Plasma Clot Lysis Time

**DOI:** 10.1371/journal.pone.0083151

**Published:** 2013-12-30

**Authors:** Zelda de Lange, Dingeman C. Rijken, Tiny Hoekstra, Karin R. Conradie, Johann C. Jerling, Marlien Pieters

**Affiliations:** 1 Centre of Excellence for Nutrition, North-West University, Potchefstroom, South Africa; 2 Department of Haematology, Erasmus University Medical Centre, Rotterdam, The Netherlands; 3 Department of Clinical Epidemiology, Leiden University Medical Centre, Leiden, The Netherlands; Maastricht University Medical Center, The Netherlands

## Abstract

Data on genetic and environmental factors influencing PAI-1 levels and their consequent effect on clot lysis in black African populations are limited. We identified polymorphisms in the promoter area of the PAI-1 gene and determined their influence on PAI-1_act_ levels and plasma clot lysis time (CLT). We also describe gene-environment interactions and the effect of urbanisation. Data from 2010 apparently healthy urban and rural black participants from the South African arm of the PURE study were cross-sectionally analysed. The 5G allele frequency of the 4G/5G polymorphism was 0.85. PAI-1_act_ increased across genotypes in the urban subgroup (p = 0.009) but not significantly in the rural subgroup, while CLT did not differ across genotypes. Significant interaction terms were found between the 4G/5G polymorphism and BMI, waist circumference and triglycerides in determining PAI-1_act_, and between the 4G/5G polymorphism and fibrinogen and fibrinogen gamma prime in determining CLT. The C428T and G429A polymorphisms did not show direct relationships with PAI-1_act_ or CLT but they did influence the association of other environmental factors with PAI-1_act_ and CLT. Several of these interactions differed significantly between rural and urban subgroups, particularly in individuals harbouring the mutant alleles. In conclusion, although the 4G/5G polymorphism significantly affected PAI-1_act_, it contributed less than 1% to the PAI-1_act_ variance. (Central) obesity was the biggest contributor to PAI-1_act_ variance (12.5%). Urbanisation significantly influenced the effect of the 4G/5G polymorphism on PAI-1_act_ as well as gene-environment interactions for the C428T and G429A genotypes in determining PAI-1_act_ and CLT.

## Introduction

The prevalence of cardiovascular disease (CVD) has increased significantly in developing countries, and will continue to do so in the future [1]. The increase in morbidity and mortality from CVD is also seen in the black population of South Africa [2], [3], particularly as a result of urbanisation, which is the change in lifestyle patterns brought about by the on-going migration from rural to urban settings. One of the factors that have repeatedly been shown to be associated with an increased risk of CVD is increased levels of plasminogen activator inhibitor type-1 (PAI-1) [4], [5]. PAI-1 can contribute to the development of CVD through several mechanisms, including influencing plaque formation as well as being a major inhibitor of blood clot lysis [6], [7]. The speed with which the body can lyse clots, often reported as lysis time, has been linked to CVD [8]. This reflects an individual's fibrinolytic potential and can be measured with the use of global fibrinolytic assays. PAI-1 antigen (PAI-1_ag_) levels have been found to explain a large portion (24%) of the variance in clot lysis time (CLT) in a white population [9], while PAI-1 activity (PAI-1_act_) explained 27% of CLT variance in the black African PURE population [10].

PAI-1 levels have previously been shown to be consistently lower in black African and African American populations than in populations of European decent [11]–[16]. However, as a result of urbanisation and the increased prevalence of various CVD risk factors associated with it, PAI-1 levels in urbanised black South Africans are increasing [16], [17]. Environmental factors known to influence PAI-1 differ significantly between rural and urban settings [17].

PAI-1 levels are influenced by both environmental and genetic factors. There is, however, not much information available in the literature regarding the genetic determinants of PAI-1 levels, especially in black Africans. One of the most extensively studied genetic polymorphisms known to influence PAI-1 levels is the 4G/5G polymorphism [18], [19]. This is a single base-pair insertion/deletion polymorphism in the promoter area of the PAI-1 gene [18], [20]. The results of a recent large genome-wide association study (GWAS) meta-analysis showed that a SNP (rs2227631), considered a proxy SNP for the 4G/5G polymorphism, was strongly associated with PAI-1 levels [21]. High PAI-1 levels have been found in participants who are homozygous for the 4G allele, intermediate levels in heterozygous participants and low PAI-1 levels in 5G homozygotes [12], [22], [23]. The 4G/5G polymorphism is, furthermore, considered to be a response polymorphism. This implies that the difference in PAI-1 levels between 4G and 5G becomes more obvious in the presence of disease and/or environmental factors which stimulate PAI-1 expression [24]. A limited number of studies in African Americans showed a higher prevalence of the 5G allele than in whites [12], [25]. Much less data is available for continental African populations. To date, only three small-scale studies have been performed in black African individuals, showing an even higher prevalence of the 5G allele than in African Americans [15], [26], [27].

The aim of this study was therefore to identify genetic polymorphisms in the promoter area of the PAI-1 gene and to determine their effect on PAI-1_act._ In addition we investigated whether these possible differences in PAI-1_act_ levels translated into altered global fibrinolytic potential (plasma clot lysis time). Owing to large differences in environmental factors between the rural and urban setting, we also aimed to investigate the effect of gene-environment interactions (e.g. BMI, blood lipids) on PAI-1_act_ and CLT and whether these interactions were influenced by urbanisation which can be viewed as a specific combination/grouping of environmental factors.

## Methods

### Study population

Participants were recruited to take part in the South African arm of the international PURE study. This is a large-scale cohort study that tracks changing lifestyles, risk factors and chronic disease, using periodic standardised data collection in rural and urban areas of 17 countries in transition over 12 years [28], [29]. The data reported here are from the baseline data of 2010 randomly selected participants from well-established rural and urban study sites in the North West Province of South Africa, collected over a twelve-week period in 2005. Details regarding the selection process and randomisation are reported elsewhere [10], [17]. In short, apparently healthy black South African men and women between the ages of 35 and 65 years were eligible to participate. Use of chronic medication for non-communicable diseases and/or any self-reported acute illness were bases for exclusion. Equal numbers of rural and urban participants were included.

### Ethics statement

The Ethics Committee of the North-West University, South Africa, approved this study. The study procedure was explained to participants in their home language, after which participants signed informed consent forms and the study commenced. All data were treated confidentially and all analyses were performed with coded data.

### Blood collection and laboratory analysis

Fasting blood samples were collected between 08:00 and 11:00. For the analysis of the lipid profile, blood was collected in tubes without anticoagulant - in EDTA tubes for the determination of glycosylated haemoglobin (HbA1c) and total homocysteine, and in fluoride tubes for glucose determination. For the determination of PAI-1_act_, fibrinogen concentration, fibrinogen gamma prime, plasma fibrinolytic potential and for DNA isolation, blood was collected in citrate tubes. Samples were centrifuged at 2000× g for 15 minutes at 10°C within 30 minutes of collection. Aliquots were frozen on dry ice, stored in the field at −18°C and then, after 2–4 days, at −82°C until analysis.

Methods for the measurement of fibrinogen, lipids, HbA1c, total homocysteine and plasma glucose have been described previously [17]. PAI-1_act_ was measured using an indirect enzymatic method (Spectrolyze PAI-1, Trinity Biotech, Bray, Ireland). A modified Clauss method (Multifibrin U-test, Dade Behring, Deerfield, IL, USA) on the Dade Behring BCS coagulation analyser was used to determine fibrinogen concentrations. Fibrinogen gamma prime was determined with an enzyme-linked immunosorbent assay (ELISA) method using a 2.G2.H9 mouse monoclonal coating antibody against the human gamma prime sequence from Santa Cruz Biotechnology (Santa Cruz, USA) for antigen capture and a goat polyclonal HRP-conjugated antibody against human fibrinogen from Abcam for development (Antibody 7539, Cambridge, USA) [30]. Plasma fibrinolytic potential using tissue-factor-induced plasma clots, lysed by exogenous tPA, was measured using the method of Lisman *et al.* [8], validated by Talens *et al.* [31] with slightly modified tissue factor and tPA concentrations in order to obtain comparable CLTs of about 60 min (intra-assay CV = 3.6%, between plate CV = 4.5%). Final concentrations were tissue factor (125× diluted – an estimated final concentration of 59 pM [32]; Dade Innovin, Siemens Healthcare Diagnostics Inc., Marburg, Germany), CaCl_2_ (17 mmol/l), tPA (100 ng/ml; Actilyse, Boehringer Ingelheim, Ingelheim, Germany) and phospholipid vesicles (10 µmol/l; Rossix, Mölndal, Sweden). CLT was defined as the time from the midpoint of the transition of clear to maximum turbidity, which is representative of clot formation, to the midpoint in transition from maximum turbidity to clear, which represents the lysis of the clot [8]. CLT and PAI-1_act_ could be determined for only 1802 and 1913 participants respectively, owing to inadequate sample volume and/or haemolysis of some samples. Baseline characteristics of these participants did not differ from those of the total group.

### PAI-1 promoter area sequencing and genotyping

Three overlapping fragments covering the promoter area from nucleotide position 100 768 248–100 770 521 (according to the NCBI database NP_000593.1) were sequenced in a subgroup of 25 randomly chosen participants and used as a representative group of the study population. PCR products were purified and bi-directionally sequenced using the BigDye®Terminator v3.1Cycle Sequencing kit (AppliedBiosystems, CA, USA) (Table S1). After this, capillary electrophoresis was performed by the Central Analytical Facility of the Stellenbosch University on a 3130*xl* GeneticAnalyser (AppliedBiosystems) [33].

Sequences were aligned to a reference sequence (NT_007933.15) using BioEdit v7 and were manually checked for known and novel polymorphisms. Seventeen polymorphisms were observed (Table S2). After linkage disequilibrium analysis and exclusion of two SNPs in linkage with the 4G/5G polymorphism, the polymorphisms with the highest frequencies, the 4G/5G polymorphism (rs1799889), located at chromosome position 100769710, and two SNPs located next to each other, at chromosome position 100768428 and 100768429 (rs36228614) were selected for analysis in the total PURE population. The novel polymorphism at position 100768428 will be referred to as C428T, and the polymorphism next to it as G429A. This SNP has been previously reported, but no data exists in the literature for this SNP. The 25 participants from the subgroup were used as controls in further analysis.

The 4G/5G-polymorphism was genotyped using TaqMan-based assays, as previously described [34], using the Biorad IQ5 real-time polymerase chain reaction (PCR) machine (Bio-Rad, Hercules, USA). A final concentration of 1.2 µM of each primer was used and 0.4 µM of each probe at an annealing temperature of 63°C.

The double nucleotide polymorphism C428T-G429A was genotyped by using a TaqMan-based assay using the BioRad IQ5 real-time PCR machine. A multiplex real-time PCR reaction was then performed, using four different probes (Table S3). A final concentration of 0.4 µM of each primer and 0.2 µM of each probe was used. The annealing temperature for the reaction was optimised at 62.4°C. Sequences are available in Table S3.

### Statistical analysis

Data were analysed with the computer software package Statistica (Statsoft Inc., Tulsa Oklahoma, USA). A p-value of 0.05 or less was regarded as statistically significant. Normally distributed data are reported as means (95% confidence interval or standard deviation). Data that were not normally distributed were log-transformed to improve normality and are reported as geometric mean (95% confidence interval). Owing to the large number of participants who had PAI-1_act_ values of 0 as calculated from the standard curve of the assay, a value of 1 was added to all PAI-1_act_ values before log transformation. This was subtracted again when reporting the data. Forward stepwise multiple regression was used to determine the contribution of various traditional CVD risk factors to PAI-1_act_ variance. Mean differences with 95%CI and t-tests were used when comparing differences between two groups. One-way analysis of variance (ANOVA) and Tukey's Honest Significant Difference post-hoc tests were used when comparing more than two groups. To investigate possible gene-environment interactions between PAI-1 genotypes and other factors and whether these interactions differed for rural and urban groups, interaction terms were entered into an analysis of co-variance (ANCOVA) with full factorial analysis. In addition, regression slopes were compared. For these interaction analyses, if sample sizes were too small, the homozygous mutant genotype groups were combined with the heterozygous group, i.e. 5G/5G compared with the 4G/X allele group, CC compared with the T/X group, and GG compared with the A/X group. Analyses of the C428T and G429A SNPs combined did not yield different results from when analysed separately (data not reported).

## Results

### PAI-1_act_ and CLT across PAI-1 genotypes

General population characteristics of the South African PURE population have been described previously [10], [17] and are included in Table S4. The distribution of all three genotype classes of each of the polymorphisms investigated adhered to the assumptions of Hardy Weinberg equilibrium. For the three polymorphisms we investigated, the linkage disequilibrium in the total study population between polymorphisms 4G/5G and C428T was: D' 1.0, r^2^ = 0.007; between 4G/5G and G429A D' 0.72, r^2^ = 0.011; and between C428T and G429A D' 0.72, r^2^ = 0.003. Because of the low r^2^, we report results for the three SNPs separately. The allele frequencies of the three polymorphisms are presented in Table 1.

**Table 1:** PAI-1_act_ levels and clot lysis time of PAI-1 promoter area polymorphisms.

Polymorphism		Allele Freq.	N (%)	PAI-1_act_ (U/ml)			CLT (min)		
				Total group	Urban	Rural	Total group	Urban	Rural
4G/5G:	5G/5G	5G: 0.85	1304 (72.5)	3.35 (3.13-3.58)*	3.93 (3.56–4.32)* †	2.90 (2.64-3.18)*	57.0 (56.4-57.6)	57.1 (56.1-58.1)	57.0 (56.2-57.8)*
				(n = 1302)	(n = 602)	(n = 700)	(n = 1223)	(n = 557)	(n = 666)
	4G/5G		448 (24.9)	3.90 (3.48-4.37)*	4.87 (4.20-5.64)*	2.95 (2.47-3.50)†	57.5 (56.4-58.6)	58.3 (56.7-59.9)	56.6 (55.1-58.1)†
				(n = 446)	(n = 243)	(n = 203)	(n = 426)	(n = 229)	(n = 197)
	4G/4G	4G: 0.15	47 (2.61)	5.87 (4.10-8.25)*	5.97 (3.45-9.92)†	5.74 (3.3.46-9.18)* ^†^	60.5 (55.8-65.2)	58.5 (51.6-65.4)	63.1 (56.3-69.8)* ^†^
				(n = 47)	(n = 26)	(n = 21)	(n = 43)	(n = 24)	(n = 19)
ANOVA p-value				p<0.001	p = 0.02	p = 0.03	p = 0.12	p = 0.40	p = 0.04
ANCOVA p-value#				(p = 0.0003)	(p = 0.009)	(p = 0.06)	(p = 0.15)	(p = 0.39)	(p = 0.12)
C428T:	CC	C: 0.96	1743 (92.3)	3.59 (3.39-3.80)	4.25 (3.82-4.60)	3.02 (2.78-3.28)	57.3 (56.7-57.8)	57.5 (56.7-58.3)	57.1 (56.3-57.8)
				(n = 1739)	(n = 861)	(n = 878)	(n = 1640)	(n = 801)	(n = 839)
	CT/TT	T: 0.04	145 (7.7)	3.06 (2.48-3.73)	4.05 (2.98-5.40)	2.44 (1.83-3.18)	57.4 (55.6-59.2)	58.0 (54.8-61.3)	56.9 (54.8-59.0)
				(n = 144)	(n = 62)	(n = 82)	(n = 136)	(n = 57)	(n = 79)
ANOVA p-value				p = 0.14•	p = 0.75•	p = 0.15•	p = 0.92•	p = 0.74•	p = 0.90•
G429A:	GG	G: 0.90	1528 (80.9)	3.61 (3.40-3.84)	4.43 (4.07-4.81)	2.92 (2.67-3.19)	57.3 (56.7-57.9)	57.6 (56.7-58.4)	57.0 (56.2-57.8)
				(n = 1524)	(n = 760)	(n = 764)	(n = 1443)	(n = 713)	(n = 730)
	GA /AA	A: 0.10	360 (19.1)	3.28 (2.88-3.72)	3.44 (2.81-4.17)	3.15 (2.66-3.71)	57.3 (56.1-58.4)	57.4 (55.4-59.3)	57.2 (55.7-58.6)
				(n = 359)	(n = 163)	(n = 196)	(n = 333)	(n = 145)	(n = 188)
ANOVA p-value				p = 0.18•	p = 0.02•	p = 0.45•	p = 0.97•	p = 0.86•	p = 0.85•

PAI-1_act_ data reported as geometric means (95% CI), normally distributed data (CLT) reported as mean (95% CI). PAI-1_act_: Plasminogen activator inhibitor type-1 activity; CLT: clot lysis time.

Values with the same symbol differed significantly between genotypes for the total, urban and rural groups respectively.

^#^ p-values after adjustment for BMI;

p-value reported for CC vs CT and TT groups combined for C428T, and GG vs GA and AA groups combined for G429A because of small sample size in homozygous mutant groups.

The geometric mean PAI-1_act_ level of the South African PURE population was 3.55±1.60 (median 4.26 (1.27–7.92)) U/ml. Data for PAI-1_act_ levels and CLT across genotypes are presented for the total population as well as for the urban and rural participants separately in Table 1. PAI-1_act_ levels for the total population and for the urban subgroup differed significantly across the 4G/5G genotypes, where 5G homozygous participants presented with the lowest PAI-1_act_ levels and 4G homozygous participants with the highest PAI-1_act_ levels, while heterozygous participants had intermediate PAI-1_act_ levels. In the rural group, PAI-1_act_ was higher in the 4G/4G group only. BMI, however, differed across genotypes in the rural group (5G/5G: 22.6, 4G/5G: 21.3 and 4G/4G: 25.1 kg/m^2^ p = 0.008), but not in the urban group (5G/5G: 23.4, 4G/5G: 23.3 and 4G/4G: 25.5 kg/m^2^ p = 0.83). Because of the known relationship between BMI and PAI-1 levels, we adjusted for BMI. After this adjustment, differences between PAI-1_act_ levels across the genotypes remained in the urban subgroup but had only borderline significance in the rural group (p = 0.06). No significant differences in clot lysis times between the genotype subclasses of the 4G/5G polymorphism were detected in the total population (p = 0.12) or in the urban group (p = 0.40). However, in the rural participants, those who had the homozygous 4G genotype had borderline significantly longer CLT than the homozygous 5G and heterozygous genotypes (63.1 vs 57.0 and 56.6 minutes, p = 0.04). Again after the adjustment for BMI, this significance disappeared.

Because the homozygous mutant genotype group (TT) of C428T was too small (n = 2) to include in further analysis as a separate group, it was combined with the CT heterozygous group. Neither PAI-1_act_ levels nor clot lysis times differed significantly between the homozygous C or T-allele group of C428T for the total population or for the rural and urban subgroups.

For the G429A SNP, the homozygous mutant (AA genotype) (n = 27) was also combined with the GA genotype for further analysis. No significant differences in PAI-1_act_ levels or clot lysis times were observed between the GG and A-allele genotypes in the total, rural or urban groups, except for PAI-1_act_, which was higher in the GG-genotype than in the group carrying the A-allele in the urban subgroup.

### Associations of cardiovascular disease risk factors with PAI-1_act_

The contributions of various traditional CVD risk factors to PAI-1_act_ variance in this population were determined using forward stepwise multiple regression. Risk factors included in the regression model were: age, sex, waist circumference, triglycerides, HDL-cholesterol, LDL-cholesterol, HbA1c, total homocysteine, fibrinogen, fibrinogen gamma prime, blood pressure, smoking and 4G/5G genotype. These factors explained 24% of the variance in PAI-1_act_ in this population. Waist circumference explained most of the variance with 12.5%, sex, triglyceride and fibrinogen concentration explained 5%, 4% and 1% respectively, while the 4G/5G polymorphism, fibrinogen gamma prime, blood pressure, smoking and HDL-cholesterol each explained less than 1%. Age, LDL-cholesterol, HbA1c and tHcy did not enter the model. Associations of CLT and CVD risk factors have been reported in De Lange *et al.* [10].

### Gene-environment interactions

Next we investigated the possibility of gene-environment interactions on PAI-1_act_ levels and CLT using the CVD risk factors that were significantly associated with PAI-1 and CLT. Results are presented for the interactions found for the 4G/5G polymorphism with PAI-1_act_ and CLT. Results reporting interactions of the C428T and G429A polymorphisms with PAI-1_act_ and CLT are available in Supporting Information S1 and Table S5.

When the participants were divided according to BMI, PAI-1_act_ differed across the genotypes of the 4G/5G polymorphism in individuals in the normal, overweight and obese BMI range, but not in underweight participants (BMI <18.5 kg.m^2^), and in participants with central adiposity but not in participants with normal waist circumferences (Table 2). CLT did not differ across the genotypes in any of the BMI categories, but tended to be longer in the 4G allele carriers in participants with central obesity. Distribution of the 4G/5G genotypes were similar in individuals with a BMI of <25 kg/m^2^ and individuals with a BMI of >25 kg/m^2^ (BMI <25 kg/m^2^: 5G/5G 73%; 4G/5G 25%; 4G/4G/ 2%: BMI >25 kg/m^2^: 5G/5G 73%; 4G/5G 23%; 4G/4G/ 3.7%). Furthermore, there was no difference in genotype distribution between individuals with abdominal obesity and those with normal waist circumference (abdominal obesity: 5G/5G 70%; 4G/5G 26%; 4G/4G/ 4%: normal waist circumference: 5G/5G 73%; 4G/5G 24%; 4G/4G/ 2.3%). There was also no difference in BMI and waist circumference (WC) across the 4G/5G genotypes (data not shown) in the total study population.

**Table 2:** PAI-1_act_ and CLT across BMI and waist circumference categories for 5G/5G participants and those harbouring the 4G genotype

			PAI-1_act_ (U/ml)			CLT (minutes)		
		N	5G/5G	4G-allele	Mean difference (95% CI)	5G/5G	4G-allele	Mean difference (95% CI)
			Mean* (95% CI)	Mean* (95% CI)		Mean (95% CI)	Mean (95% CI)	
BMI (kg/m^2^):	<18.5	319	1.89 (1.56-2.26)	1.64 (1.18-2.21)	0.24 (-0.14-0.62)	50.7 (49.2-52.2)	50.9 (48.6-53.2)	0.24 (-2.48-2.95)
	18.5-24.9	731	2.65 (2.37-2.94)	3.59 (3.03-4.22)	0.94 (0.69-1.18)	54.9 (54.0-55.8)	56.1 (54.5-57.7)	1.22 (-0.59-3.02)
	25-29.9	298	4.83 (4.23-5.49)	6.22 (4.95-7.75)	1.39 (1.05-1.74)	60.7 (59.4-61.9)	61.6 (58.8-64.4)	0.92 (-2.11-3.95)
	≥30	353	5.52 (4.87-6.25)	7.55 (6.12-9.28)	2.03 (1.69-2.37)	64.2 (63.1-65.3)	65.1 (63.0-67.3)	0.91 (-1.52-3.33)
Waist circumference:	Normal WC	1422	2.89 (2.67-3.12)	3.14 (2.76-3.55)	0.25 (-0.07-0.43)	55.3 (54.6-56.0)	55.3 (54.2-56.5)	0.06 (-1.24-1.36)
	Centrally obese	356	5.59 (5.22-6.62)	9.29 (7.78-11.1)	3.41 (3.12-3.70)	64.4 (63.3-65.5)	66.7 (64.3-69.2)	2.33 (-0.33-4.99)

Geometric mean; PAI-1_act_: Plasminogen activator inhibitor type-1 activity; CLT: clot lysis time; BMI: Body mass index; WC: Waist circumference; Normal WC: <80cm for women and <94cm for men; Central obesity, ≥80cm for women and ≥94cm for men.

The 4G/5G polymorphism had significant interactions with WC (p = 0.001), BMI (p = 0.006) and triglyceride concentration (p = 0.04) in determining PAI-1_act_ (Table 3). In participants with the 4G allele, there was a greater increase in PAI-1_act_ with an increase in WC, BMI and triglycerides than in 5G/5G homozygotes. The 4G/5G polymorphism had significant interactions with fibrinogen (p = 0.005) and fibrinogen gamma prime (p = 0.04) concentration in determining CLT (Table 3). In participants with the 4G allele, there was a greater increase in CLT with an increase in fibrinogen and fibrinogen gamma prime concentration than in 5G/5G homozygotes. Urbanisation did not influence these gene-environment interactions.

**Table 3:** PAI-1_act_: plasminogen activator inhibitor-1 activity; BMI: body mass index; CLT: clot lysis time.

			5G/5G		4G allele	
Interaction		Interaction p-value*	N	Slope (95% CI)	N	Slope (95% CI)
PAI-1_act_:	Waist circumference (cm)	0.001	1289	1.89 (1.57; 2.21)	489	2.92 (2.42; 3.43)
	BMI (kg/m^2^)	0.006	1241	1.15 (0.96; 1.34)	460	1.66 (1.35; 1.97)
	Triglycerides (mmol/l)	0.04	1259	0.56 (0.46; 0.66)	481	0.78 (0.60; 0.95)
CLT:	Fibrinogen (g/L)	0.005	1149	2.69 (1.55; 3.82)	425	5.94 (3.83; 8.06)
	Fibrinogen gamma prime (g/L)	0.04	1173	6.78 (5.69; 7.88)	444	9.04 (7.06; 11.0)

PAI-1_act_: plasminogen activator inhibitor-1 activity; BMI: body mass index; CLT: clot lysis time.

• Slope: regression line obtained by plotting PAI-1_act_ or CLT (y-axis) against environmental factors (x-axis).

p-values indicate significance of the interaction term of the 4G/5G genotype and environmental factor in an ANCOVA.

The genotypes of the C428T and G429A polymorphisms also had significant interactions with various CVD risk factors in determining PAI-1_act_ and CLT (Table S5). In general it seems that, for both polymorphisms, differences in gene-environment interactions were observed between rural and urban participants in individuals harbouring the respective mutant alleles, while no rural-urban differences were present in participants homozygous for the respective wild type alleles.

## Discussion

This study provides the first population-based data for genotype frequencies of the 4G/5G polymorphism in a large black African population. To date there have been only three small-scale studies and our data indicate an even higher prevalence of the 5G allele than previously reported for African Americans. In addition we aimed to identify other factors contributing to PAI-1_act_ variance in Africans by identifying other polymorphisms in the PAI-1 promoter area and determining their relation to PAI-1_act_ levels and CLT. Lastly we demonstrated that gene-environment interactions between the three investigated polymorphisms and several traditional CVD risk factors significantly affected PAI-1_act_ and CLT, and that level of urbanisation (rural or urban), representing two different sets of specific combinations of environmental factors, significantly influenced phenotypic expression of these polymorphisms.

### PAI-1_act_ and CLT across PAI-1 genotypes

In this large black African population we found a high prevalence of the 5G/5G genotype (72.5%) of the 4G/5G polymorphism. These results agree with the results of two small-scale studies conducted in black South African participants, in which the 5G homozygous genotype was present in 76% and 77.6% of the populations in comparison with 36% and 19.3% in white South Africans [15], [26]. The 5G allele frequency (0.85) was higher than that reported for African American, (0.74 and 0.72), and white populations (0.38 and 0.28) [12], [25].

In agreement with results from other studies, we also found PAI-1_act_ levels of the 4G homozygous genotype to be higher than those of the 4G/5G heterozygous and 5G homozygous genotypes, with the 4G/5G genotype group presenting with intermediate PAI-1_act_ levels in the overall population [12], [22], [23]. Interestingly, this difference in PAI-1_act_ levels between genotypes of the 4G/5G polymorphism was much more pronounced in the urban than in the rural subgroup, suggesting that urbanisation-dependent stimuli might modulate gene expression through an interaction with the 4G/5G polymorphism. Future studies identifying these stimuli are warranted to confirm this hypothesis. Despite increased PAI-1_act_ levels in both the rural and urban subgroups in the 4G/4G genotype, this change did not translate to longer CLTs.

Neither PAI-1_act_ nor CLT differed across the novel C428T or the G429A genotype except in the urban subgroup, where homozygous wildtype participants had significantly higher PAI-1_act_ levels than the A-allele group. Although the G429A polymorphism has been previously identified, no information is available in the literature regarding its effect on PAI-1 levels. A possible reason for the apparent lack of effect of these two SNPs may be that the SNPs were not present within a known transcription factor binding site (as determined with the UCSC genome browser using the TFBS conserved and ENCODE regulation tracks).

### Associations of cardiovascular disease risk factors with PAI-1_act_

The 4G/5G polymorphism explained less than one percent of the variance of PAI-1_act_ in this population. The main contributor to PAI-1_act_ variance in this population was waist circumference, followed by sex and triglyceride concentration. This is in agreement with Verschuur *et al.* [35], who found obesity to be a more important determinant of PAI-1 levels than genetic variation in the PAI-1 promoter area. It is also possible, however, that PAI-1 levels can be influenced by polymorphisms outside the PAI-1 gene. Huang *et al.* [21], for example recently found SNPs in the ARNTL and the PPARG genes to have genome-wide significant association with circulating PAI-1 levels.

### Gene-environment interactions - 4G/5G polymorphism

The relationship between PAI-1 levels, the 4G/5G genotype and BMI, although extensively studied, remains controversial. Genotype-specific differences in PAI-1_act_ levels were present in all BMI ranges in our study population, except for BMI <18.8 kg/m^2^, whereas in white populations, they have sometimes been observed in obese individuals only [36]–[38], while in other studies [35] they have appeared in lean individuals only. In agreement with results from Sartori *et al.* [38], these differences were also present in individuals with central obesity but not in those with a normal waist circumference. We additionally found significant interactions of the 4G/5G genotypes with waist circumference and BMI in determining PAI-1_act_ levels in this apparently healthy population, with PAI-1_act_ increasing more rapidly with increasing BMI and WC in the 4G allele group. These results are in agreement with McCormack *et al.* [39], who investigated these associations in Pima Indians. Ossei-Gerning *et al.* [23], however, found the association between BMI and PAI-1 to be stronger in the 5G/5G genotype in a group of 453 white patients with a history of myocardial infarction. *In vitro* studies aiming at elucidating possible mechanisms for this interaction, found no effect of genotype on promoter activity in HepG2 or BEAC cells [35], nor did it influence the adipose secretion rate of PAI-1 [40]. With the 4G/5G polymorphism being a response polymorphism, ethnic differences as well as differences in study population characteristics (healthy vs diseased), probably contribute to these discrepancies in the literature.

The relationship between PAI-1_act_ and triglyceride levels was also found to be influenced by the 4G/5G genotype in our study population, with PAI-1_act_ increasing more rapidly with increasing triglycerides in the 4G allele group. Similar associations were found in studies investigating CVD patients [23], [41], [42], although in studies investigating healthy white individuals, genotype was not found to influence this relationship [22], [43]. A possible explanation for the observed 4G/5G–triglyceride interaction is that the activity of a VLDL-response element in the PAI-1 promoter area was found to be influenced by the 4G/5G polymorphism located adjacent to and upstream of the binding site of the VLDL-inducible transcription factor [44].

As yet, it is still unclear whether PAI-1 plays a role in the evolution of obesity and whether the 4G/5G polymorphism could possibly contribute to this. While some case-control studies found differences in genotype distribution between obese and non-obese participants [45], [46], others did not [38]. Our data, providing the first population-based evidence, indicated that there were no differences in genotype distribution between individuals of normal weight and those who were overweight/obese. Additionally, BMI and WC did not differ across the genotypes. This is in agreement with Gardemann *et al.* [47] but in contradiction to Hoffsted *et al.* [45], who found the prevalence of obesity to be twice as high in the 4G allele as in the 5G allele group. In an *in vitro* experiment, Demiralp *et al.* [48] suggest that overexpression of PAI-1 in 3T3-L1 cells can increase adipocyte differentiation and that the 4G allele was significantly more active than the 5G allele in driving PAI-1 gene transcription, in this way contributing to adipogenesis. More evidence from population-based studies, comparing different ethnicities is required, however, to determine the role of PAI-1 and its gene regulation in obesity. Genome wide association studies (GWAS) have shown that various genes influence obesity [49], [50], but, to our knowledge no GWAS have been published investigating the influence of the polymorphisms we investigated and their associations with obesity.

We found significant interactions of the 4G/5G genotypes with fibrinogen and fibrinogen gamma prime in determining CLT. Fibrinogen gamma prime was included in this investigation as it has been shown to affect clot structure, which may influence lysis [51]. In participants with the 4G allele, there was a greater increase in clot lysis time with an increase in fibrinogen and fibrinogen gamma prime than in 5G/5G homozygotes. Olman *et al.* [52] identified an AP-1-like DNA element as important in transcriptional control in the PAI-1 gene. According to Olman *et al.* [52], a negative regulatory feedback loop is initiated, wherein D-dimers, formed after fibrin degradation, generate a signal that results in PAI-1 transcription through the AP-1 expression element, inhibiting fibrinolysis. The influence of increasing fibrinogen and fibrinogen gamma prime concentrations on clot lysis in the present study may, therefore, amongst other reasons, be through the increased generation of D-dimer. If the AP-1 expression element enhances PAI-1 mRNA in the 4G but not the 5G allele, as has been demonstrated for interleukin-1 [18] and VLDL, it could at least in part explain the observed gene-environment interactions. The enhanced transcription of PAI-1 could consequently result in prolonged CLT. Additional research is required, however, to test this hypothesis.

### C428T and G429A polymorphisms

Despite there being no direct relationship between these polymorphisms and PAI-1_act_ and CLT, we did find evidence that the association of other environmental factors with PAI-1_act_ and CLT was influenced by these genotypes. Additionally, many, but not all, of the gene-environment interactions differed consistently between rural and urban participants in subjects harbouring the mutant alleles, but not in subjects harbouring the most common genotype. These data suggest that significant differences in environmental factors in urban and rural living conditions could potentially influence phenotypic expression of the investigated SNPs in subjects harbouring the mutant variants and that these individuals may be more sensitive towards environmental factors than individuals harbouring the respective common genotypes.

In conclusion, this black African population had a high prevalence of the 5G allele (0.85). The 4G/5G polymorphism significantly affected PAI-1_act_ but not CLT. The polymorphism contributed, however, to less than 1% in the PAI-1_act_ variance. Obesity had a much more pronounced effect on PAI-1_act_ than the measured polymorphisms. The C428T and G429A SNPs had no direct association with either PAI-1_act_ or CLT. There were significant gene-environment interactions for all three polymorphism investigated in determining PAI-1_act_ and CLT. Urbanisation significantly affected the phenotypic expression of the 4G/5G polymorphism, with PAI-1_act_ showing larger differences across the 4G/5G genotypes in the urban community than in the rural community. Urbanisation additionally influenced gene-environment interactions, with differences between rural and urban participants observed particularly in participants harbouring the minor alleles of the C428T and G429 polymorphisms. From these results it seems clear that environmental factors and combinations thereof, such as specific combinations present in a rural or urban environment, significantly influence phenotypic expression of genes. This should be taken into consideration when developing treatment modalities addressing CVD risk factors such as PAI-1 and hypofibrinolysis in communities in transition.

## Supporting Information

Table S1 **Primers used to sequence PAI-1 promoter area.**
(DOC)

Table S2 **Polymorphisms found in the subgroup of n = 25 of the PURE population.** * rs numbers not available. • In n = 22 individuals the genotypes for polymorphisms number 7 and 8 were C/C and A/A respectively. In n = 3 individuals genotype status could not be determined due to homopolymeric interference with sequencing electropherograms. ^†^ dbSNP version 137 was used to determine nucleotide positions.
(DOC)

Table S3 **Primers and probes used for genotyping of PAI-1 polymorphisms.**
(DOC)

Table S4 **Characteristics of total study population, urban and rural participants.** Data reported as: mean ± std; *Plasma PAI-1_act_ reported as geometric mean ± std; ^‡^ Significant difference between men and women; M: male; F: female; HIV+human immunodeficiency virus-infected; LDL: low density lipoprotein; HDL: High density lipoprotein; CRP: c-reactive protein; CLT: clot lysis time.
(DOC)

Table S5 **Significant gene-environment interactions for the C428T and G429A genotypes in determining PAI-1_act_ and CLT - the effect of urbanisation.** PAI-1_act_: plasminogen activator inhibitor −1 activity; HDL-chol: high density lipoprotein cholesterol; tHcy: total homocysteine; CLT: clot lysis time; BMI: body mass index; LDL-chol: low density lipoprotein cholesterol; SBP: systolic blood pressure; R: rural; U: urban. • Slope: regression line obtained by plotting PAI-1_act_ or CLT (y-axis) against environmental factors (x-axis). * p-values indicate significance of interaction term between SNP and environmental factor in an ANCOVA. Where data are reported for rural and urban groups separately, urbanisation influenced the gene-environment interaction. ^▪^ Rural and urban slopes differed significantly.
(DOC)

Information S1 (DOC)
