# Supplementary material for: In Black South Africans from Rural and Urban Communities, the 4G/5G PAI-1 Polymorphism Influences PAI-1 Activity, but Not Plasma Clot Lysis Time
Source: PLoS One. 2013 Dec 30;8(12):e83151. doi: 10.1371/journal.pone.0083151 (PMC3875438; doi:10.1371/journal.pone.0083151)
Supplement: Table S1 — Primers used to sequence PAI-1 promoter area. (DOC) [file pone.0083151.s001.doc]

**Table S1.** Primers used to sequence PAI-1 promoter area

| Primer name | Sequence 5’ – 3’ | Design |
| --- | --- | --- |
| PAI_1_F | TTCCACCCACTGAAACTTCC | Own design |
| PAI_1_R | GATGGGAGACCGTGACAGAT | Own design |
| PAI_2_F | GGTTGCAAGCTCCCTATGAG | Own design |
| PAI_2_R | CAGCCACGTGATTGTCTAGG | Own design |
| PAI_3_F | GGGAGTCAGCCGTGTATCAT | Own design |
| PAI_3_R | AGTTCTCAGAGGTGCCTTGC | Own design |
