# Supplementary material for: In Black South Africans from Rural and Urban Communities, the 4G/5G PAI-1 Polymorphism Influences PAI-1 Activity, but Not Plasma Clot Lysis Time
Source: PLoS One. 2013 Dec 30;8(12):e83151. doi: 10.1371/journal.pone.0083151 (PMC3875438; doi:10.1371/journal.pone.0083151)
Supplement: Table S2 — Polymorphisms found in the subgroup of n = 25 of the PURE population. * rs numbers not available. • In n = 22 individuals the genotypes for polymorphisms number 7 and 8 were C/C and A/A respectively. In n = 3 individuals genotype status could not be determined due to homopolymeric interference with sequencing electropherograms. † dbSNP version 137 was used to determine nucleotide positions. (DOC) [file pone.0083151.s002.doc]

**Table S2**. Polymorphisms found in the subgroup of n=25 of the PURE population

| Number | Position on chromosome 7^†^ | rs number | Polymorphism | Frequency of polymorphism in n=25 participants | Measured in PURE population |
| --- | --- | --- | --- | --- | --- |
| 1 | Chr7:100768307 | * | C/T | n=2 | Excluded |
| 2 | Chr7:100768428 | * | T/C | n=2 | C428T included because of position next to G429A |
| 3 | Chr7:100768429 | rs36228614 | A/G | n=8 | G429A included |
| 4 | Chr7:100768545 | * | G/A | n=4 | Excluded |
| 5 | Chr7:100768600 | rs36228272 | T/C | n=1 | Excluded |
| 6 | Chr7:100768757 | rs36228273 | Insertion A | n=3 | Excluded |
| 7 | Chr7:100768974 | rs2227629 | C/T | Homopolymeric interference• | Excluded |
| 8 | Chr7:100769060 | rs2227630 | A/T | Homopolymeric interference• | Excluded |
| 9 | Chr7:100769286 | rs2227707 | C/T | n=0 | Excluded |
| 10 | Chr7:100769538 | rs2227631 | A/G | n=7 | In linkage with 11, therefore excluded |
| 11 | Chr7:100769710/1 | rs1799889 | 4G/5G | n=6 | 4G/5G included |
| 12 | Chr7:100769875 | rs2227632 | A/G | n=4 | In linkage with 11, therefore excluded |
| 13 | Chr7:100768908 | * | G/A | n=3 | Excluded |
| 14 | Chr7:100768983 | * | A/T | n=1 | Excluded |
| 15 | Chr7:100770208/7 | rs2227633 | CA Insertion/deletion | n=5 | Still under investigation |
| 16 | Chr7:100769306 | * | Insertion T | n=1 | Excluded |
| 17 | Chr7:100770458 | rs2227635 | T/C | n=0 | Excluded |

* rs numbers not available

• In n=22 individuals the genotypes for polymorphisms number 7 and 8 were C/C and A/A respectively. In n=3 individuals genotype status could not be determined due to homopolymeric interference with sequencing electropherograms.

^†^ dbSNP version 137 was used to determine nucleotide positions.
