# Supplementary material for: In Black South Africans from Rural and Urban Communities, the 4G/5G PAI-1 Polymorphism Influences PAI-1 Activity, but Not Plasma Clot Lysis Time
Source: PLoS One. 2013 Dec 30;8(12):e83151. doi: 10.1371/journal.pone.0083151 (PMC3875438; doi:10.1371/journal.pone.0083151)
Supplement: Table S3 — Primers and probes used for genotyping of PAI-1 polymorphisms. (DOC) [file pone.0083151.s003.doc]

**Table S3.** Primers and probes used for genotyping of PAI-1 polymorphisms

| Polymorphism | Name | Sequence 5’ – 3’ | Design |
| --- | --- | --- | --- |
| 4G/5G | Primer 4G5GF: | TCTTTCCCTCATCCCTGCC | Ref. 1 |
|  | Primer 4G5GR: | CCAACCTCGCCAGACAAGG |  |
|  | Probe: PAI 4C: | 5HEX/ACACGGCTGACTCCCCACGT/3BHQ_1 |  |
|  | Probe: PAI 5C: | 56-FAM/ACGGCTGACTCCCCCACGT/3BHQ_1 |  |
| C428T & G429A | Primer PAI_F: | TCCCACCCACTGAAACTTCC | Own design |
|  | Primer PAI_R: | GGTGAGCATGTAGGGCTAGACT |  |
|  | Probe PAI_TG: | 5CY5/ATCCAGACCACA**TG**GCCAAG/3lAbRQSp |  |
|  | Probe PAI_TA: | 5TexRd-XN/ACCACA**TA**GCCAAGGGCACC/3lAbRQSp |  |
|  | Probe PAI_CA: | 5HEX/TCCAGACCACA**CA**GCCAAGG/3BHQ_1 |  |
|  | Probe PAI_CG: | 56-FAM/CAGACCACA**CG**GGCCAAGG/3BHQ_1 |  |
