# Supplementary material for: In Black South Africans from Rural and Urban Communities, the 4G/5G PAI-1 Polymorphism Influences PAI-1 Activity, but Not Plasma Clot Lysis Time
Source: PLoS One. 2013 Dec 30;8(12):e83151. doi: 10.1371/journal.pone.0083151 (PMC3875438; doi:10.1371/journal.pone.0083151)
Supplement: Table S4 — Characteristics of total study population, urban and rural participants. Data reported as: mean ± std; *Plasma PAI-1act reported as geometric mean ± std; ‡ Significant difference between men and women; M: male; F: female; HIV+human immunodeficiency virus-infected; LDL: low density lipoprotein; HDL: High density lipoprotein; CRP: c-reactive protein; CLT: clot lysis time. (DOC) [file pone.0083151.s004.doc]

**Table S4.** Characteristics of total study population, urban and rural participants

| Variable |  | Total population | Urban | Rural | Mean difference (95% CI) |
| --- | --- | --- | --- | --- | --- |
|  |  | N=2010 | N=1004 | N=1006 |  |
| Age (yr) |  | 49.3 ± 10.4 | 50.2 ± 10.7 | 48.5 ± 9.93 | 1.74 (0.83-2.64) |
| Gender M/F (%) |  | 37.3 / 62.7 | 39.9 / 60.1 | 34.6 / 65.4 | 5.3 / 5.3 |
| HIV + (%) |  | 16.2 | 15.7 | 16.8 | 1.1 |
| Smoking status (%) | Never | 43.8 | 42.7 | 44.9 | 2.2 |
|  | Past | 3.80 | 3.90 | 3.80 | 0.1 |
|  | Current | 51.8 | 52.6 | 51.1 | 1.5 |
| Blood pressure (mmHg) | Systolic | 133.5 ± 24.5 | 137 ± 25.1 | 129.7 ± 23.3 | 7.61 (5.48-9.73) |
|  | Diastolic | 87.7 ± 14.5 | 89.3 ± 14.5 | 86.2 ± 14.5 | 3.12 (1.85-4.39) |
| Body mass index (kg/m^2^) |  | 24.6 ± 6.96 | 25.2 ± 7.29 | 24.2 ± 6.61 | 1.00 (0.37-1.63) |
| Waist circumference (cm) |  | 79.3 ± 13.0 | 80.6 ± 13.3 | 78.8 ± 12.6 | 1.83 (0.69-2.97) |
|  | Men | 76.3 ± 9.80 ^‡^ | 76.1 ± 9.68 ^‡^ | 76.5 ± 9.96 ^‡^ | 0.36 (-1.06-1.78) |
|  | Women | 81.7 ± 14.2 ^‡^ | 83.6 ± 14.5 ^‡^ | 80.0 ± 13.7 ^‡^ | 3.60 (2.03-5.16) |
| Serum total cholesterol (mM) |  | 5.01 ± 1.38 | 5.05 ± 1.4 | 4.96 ± 1.36 | 0.09 (-0.03-0.21) |
| Serum LDL-cholesterol (mM) |  | 2.92 ± 1.17 | 2.93 ± 1.18 | 2.92 ± 1.17 | 0.01 (-0.10-0.12) |

| Serum HDL-cholesterol (mM) |  | 1.52 ± 0.63 | 1.52 ± 0.65 | 1.52 ± 0.62 | 0.003 (-0.05-0.06) |
| --- | --- | --- | --- | --- | --- |
|  | Men | 1.58 ± 0.66 ^‡^ | 1.61 ± 0.66 ^‡^ | 1.55 ± 0.66 | 0.06 (-0.04-0.16) |
|  | Women | 1.48 ± 0.62 ^‡^ | 1.46 ± 0.63 ^‡^ | 1.50 ± 0.61 | 0.04 (-0.03-0.11) |
| Serum triglycerides (mM) |  | 1.30 ± 0.79 | 1.38 ± 0.91 | 1.21 ± 0.64 | 0.17 (0.10-0.24) |
| Plasma glucose (mM) |  | 5.02 ± 2.73 | 5.17 ± 3.7 | 4.87 ± 1.23 | 0.29 (0.04-0.54) |
| Serum CRP (mg/L) |  | 8.52 ± 12.5 | 8.70 ± 12.3 | 8.34 ± 12.6 | 0.36 (-0.77-1.48) |
| Plasma fibrinogen (g/L) |  | 3.69 ± 2.18 | 3.51 ± 2.13 | 3.86 ± 2.21 | 0.35 (0.15-0.55) |
|  | Men | 3.33 ± 2.07 ^‡^ | 3.17 ± 1.98 ^‡^ | 3.50 ± 2.15 ^‡^ | 0.33 (0.01-0.64) |
|  | Women | 3.92 ± 2.21 ^‡^ | 3.75 ± 2.20 ^‡^ | 4.06 ± 2.21 ^‡^ | 0.31 (0.05-0.57) |
| Plasma PAI-1_act_ (U/mL)* |  | 3.55 ±1.60 | 4.26 ± 1.62 | 2.96 ± 1.54 | 1.29 (-1.15-1.44) |
| CLT (min) |  | 57.3 ± 11.2 | 57.6 ± 12.0 | 57.0 ± 10.5 | 0.57 (-0.47-1.61) |
|  | Men | 52.9 ± 11.6 ^‡^ | 52.6 ± 12.4 ^‡^ | 53.3 ± 10.6 ^‡^ | 0.64 (-1.10-2.38) |
|  | Women | 59.9 ± 10.2 ^‡^ | 60.8 ± 10.5 ^‡^ | 59.0 ± 10.0 ^‡^ | 1.79 (0.60-2.99) |
| Plasma homocysteine (μM) |  | 10.3 ± 4.57 | 9.84 ± 4.26) | 10.8 ± 4.80 | 0.98 (0.56-1.39) |
|  | Men | 11.3 ± 4.47 ^‡^ | 10.4 ± 3.76 ^‡^ | 12.2 ± 4.98 ^‡^ | 1.82 (1.17-2.48) |
|  | Women | 9.78 ± 4.54 ^‡^ | 9.47 ± 4.54 ^‡^ | 10.1 ± 4.53 ^‡^ | 0.59 (0.07-1.11) |

Data reported as: mean ± std; *Plasma PAI-1_act_ reported as geometric mean ± std; ^‡^ Significant difference between men and women; M: male; F: female; HIV + human immunodeficiency virus‑infected; LDL: low density lipoprotein; HDL: High density lipoprotein; CRP: c-reactive protein; CLT: clot lysis time.
