# Supplementary material for: In Black South Africans from Rural and Urban Communities, the 4G/5G PAI-1 Polymorphism Influences PAI-1 Activity, but Not Plasma Clot Lysis Time
Source: PLoS One. 2013 Dec 30;8(12):e83151. doi: 10.1371/journal.pone.0083151 (PMC3875438; doi:10.1371/journal.pone.0083151)
Supplement: Table S5 — Significant gene-environment interactions for the C428T and G429A genotypes in determining PAI-1act and CLT - the effect of urbanisation. PAI-1act: plasminogen activator inhibitor −1 activity; HDL-chol: high density lipoprotein cholesterol; tHcy: total homocysteine; CLT: clot lysis time; BMI: body mass index; LDL-chol: low density lipoprotein cholesterol; SBP: systolic blood pressure; R: rural; U: urban. • Slope: regression line obtained by plotting PAI-1act or CLT (y-axis) against environmental factors (x-axis). * p-values indicate significance of interaction term between SNP and environmental factor in an ANCOVA. Where data are reported for rural and urban groups separately, urbanisation influenced the gene-environment interaction. ▪ Rural and urban slopes differed significantly. (DOC) [file pone.0083151.s005.doc]

**Table S5.** Significant gene‑environment interactions for the C428T and G429A genotypes in determining PAI-1_act_ and CLT - the effect of urbanisation

| Interaction | Interaction p-value* | Common genotype | | Mutant allele | |
| --- | --- | --- | --- | --- | --- |
|  |  | Slope (95% CI)• | | Slope (95% CI)• | |
|  |  | (N) | | (N) | |
| C428T |  |  |  |  |  |
| PAI-1_act_: |  |  |  |  |  |
| Triglycerides (mmol/l) | 0.04 | R: 0.62 (0.49; 0.76) | U: 0.55 (0.43; 0.67) | R: 0.98 (0.55; 1.41)^■^ | U: 0.14 (-0.34; 0.66)^■^ |
|  |  | (851) | (845) | (78) | (61) |
| HDL-chol (mmol/l) | 0.03 | R: -0.20 (-0.30; -0.04) | U: -0.30 (-0.40; -0.20) | R:0.08 (-0.27; 0.44)^■^ | U:-0.59 (-0.87; -0.31)^■^ |
|  |  | (861) | (847) | (78) | (61) |
| tHcy (µmol/l) | 0.01 | R:-0.17 (-0.33; -0.01) | U:-0.13 (-0.31; 0.06) | R: 0.30 (-0.24; 0.85)^■^ | U:-0.72 (-1.23; -0.20)^■^ |
|  |  | (863) | (826) | (82) | (59) |
| Fibrinogen gamma prime (g/L) | 0.05 | R: 0.40 (0.30; 0.51)^■^ | U:0.15 (0.02; 0.29)^■^ | R:0.07 (-0.27; 0.40) | U: 0.58 (-0.04; 1.20) |
|  |  | (838) | (775) | (77) | (55) |

| CLT: |  |  | |  | |
| --- | --- | --- | --- | --- | --- |
| BMI (kg/m^2^) | 0.01 | 19.5 (17.6; 21.4) | | 10.9 (4.45; 17.4) | |
|  |  | (1552) | | (128) | |
| G429A |  |  |  |  |  |
| PAI-1_act_: |  |  |  |  |  |
| LDL-chol (mmol/l) | 0.02 | R: 0.38 (0.23; 0.52) | U: 0.23 (0.08; 0.37) | R: -0.15 (-0.47; 0.17)^■^ | U: 0.34 (-0.01; 0.70)^■^ |
|  |  | (742) | (735) | (185) | (160) |
| Fibrinogen (g/L) | 0.002 | R: 0.003 (-0.12; 0.13) | U:0.04 (-0.08; 0.17) | R: 0.28 (0.05; 0.52)^■^ | U: -0.33 (-0.60; -0.05)^■^ |
|  |  | (731) | (693) | (189) | (143) |
| SBP (mm/Hg) | 0.03 | 0.004 (0.002; 0.006) | | -0.001 (-0.0005; 0.003) | |
|  |  | (1514) | | (355) | |
| CLT: |  |  | |  | |
| Fibrinogen (g/L) | 0.06 | 3.86 (2.77; 4.95) | | 1.44 (-0.74; 3.63) | |
|  |  | (1349) | | (306) | |
| SBP (mm/Hg) | 0.04 | -0.001 (-0.02; 0.02) | | -0.06 (-0.11; -0.01) | |
|  |  | (1433) | | (328) | |

PAI-1_act_: plasminogen activator inhibitor -1 activity; HDL-chol: high density lipoprotein cholesterol; tHcy: total homocysteine; CLT: clot lysis time; BMI: body mass index; LDL-chol: low density lipoprotein cholesterol; SBP: systolic blood pressure; R: rural; U: urban. • Slope: regression line

obtained by plotting PAI-1_act_ or CLT (y-axis) against environmental factors (x-axis). * p-values indicate significance of interaction term between SNP and environmental factor in an ANCOVA. Where data are reported for rural and urban groups separately, urbanisation influenced the gene-environment interaction. ^■^ Rural and urban slopes differed significantly.
