# Supplementary material for: In Black South Africans from Rural and Urban Communities, the 4G/5G PAI-1 Polymorphism Influences PAI-1 Activity, but Not Plasma Clot Lysis Time
Source: PLoS One. 2013 Dec 30;8(12):e83151. doi: 10.1371/journal.pone.0083151 (PMC3875438; doi:10.1371/journal.pone.0083151)
Supplement: Information S1 — (DOC) [file pone.0083151.s006.doc]

**Supporting Information S1**

*Gene‑environment interactions of the C428T and G429A polymorphisms*

The genotypes of the C428T polymorphism had significant interactions with triglyceride concentration (p=0.04), HDL-cholesterol (p=0.03), total homocysteine (p=0.01) and fibrinogen gamma prime concentration (p=0.05) in determining PAI-1_act_ levels. Differences between these gene‑environment interactions were found, however, between the rural and urban subgroups. The significant interactions are presented in Table S5 of this supplement. PAI-1_act_ increased significantly with an increase in triglyceride concentration. In participants with the homozygous common genotype, this association did not differ between rural and urban subgroups, but in participants harbouring the T-allele, the association between PAI-1_act_ and triglyceride concentration differed significantly between the rural and urban subgroups. This same pattern can be seen in participants harbouring the T‑allele for the association between PAI-1_act_ and HDL-C and tHcy, where the association between PAI-1_act_ and these factors did not differ between the rural and urban subgroups for the homozygous common genotype but differed significantly between rural and urban subgroups in participants harbouring the T-allele (Table S5). The genotypes of the C428T polymorphism showed a significant interaction with BMI (p=0.01) in determining CLT. The association between CLT and BMI was less pronounced in the participants harbouring the T‑allele than in the homozygous common genotype (Table S5).

The G429A polymorphism had significant interactions with LDL-cholesterol (p=0.02) and fibrinogen (p=0.002) in determining PAI-1_act_ levels. These interactions differed significantly between the rural and urban subgroups. As was the case for the C428T

polymorphism, there was no difference in the association of PAI-1_act_ with LDL-cholesterol and fibrinogen between the rural and urban subgroups in the homozygous common genotype participants. In the participants harbouring the A-allele, however, the association between PAI-1_act_ and LDL-cholesterol and fibrinogen differed significantly between the rural and urban subgroups. The genotypes of the G429A polymorphism showed a significant interaction with systolic blood pressure in determining PAI-1_act_ (p=0.03); this interaction was not influenced by urbanisation (Table S5).

The G429A polymorphism had significant interactions with fibrinogen (p=0.06) and systolic blood pressure (p=0.04) in determining CLT. In the homozygous common genotype group, CLT increased with an increase in fibrinogen concentration, while no association was found in the group harbouring the A-allele. On the other hand, no association was found between CLT and systolic blood pressure in the homozygous common genotype group, while CLT decreased with an increase in systolic blood pressure in the group harbouring the A-allele (Table S5).
